# Fibroblast Growth Factor-21, Leptin, and Adiponectin Responses to Acute Cold-Induced Brown Adipose Tissue Activation

**DOI:** 10.1210/clinem/dgaa005

**Published:** 2020-01-08

**Authors:** Lijuan Sun, Jianhua Yan, Hui Jen Goh, Priya Govindharajulu, Sanjay Verma, Navin Michael, Suresh Anand Sadananthan, Christiani Jeyakumar Henry, S Sendhil Velan, Melvin Khee-Shing Leow

**Affiliations:** 1 Clinical Nutrition Research Centre, Singapore Institute for Clinical Sciences, Agency for Science, Technology and Research (A*STAR) and National University Health System (NUHS), Singapore; 2 Shanghai Key Laboratory for Molecular Imaging, Shanghai University of Medicine and Health Sciences, Shanghai, China; 3 Department of Nuclear Medicine, First Hospital of Shanxi Medical University, Taiyuan, Shanxi, China; 4 Molecular Imaging Precision Medicine Collaborative Innovation Centre, Shanxi Medical University, Taiyuan, China; 5 Laboratory of Molecular Imaging, Singapore Bioimaging Consortium, Agency for Science Technology and Research (A*STAR), Singapore; 6 Singapore Institute of Clinical Sciences, Agency for Science Technology and Research (A*STAR), Singapore; 7 Department of Biochemistry, Yong Loo Lin School of Medicine, National University of Singapore (NUS), Singapore; 8 Departments of Physiology & Medicine, National University of Singapore (NUS), Singapore; 9 Cardiovascular and Metabolic Disorders Program, Duke-NUS Medical School, Singapore; 10 Lee Kong Chian School of Medicine, Nanyang Technological University (NTU), Singapore; 11 Department of Endocrinology, Tan Tock Seng Hospital (TTSH), Singapore

**Keywords:** cold-induced supraclavicular BAT activity, adiponectin, FGF21, leptin, TNFα

## Abstract

**Background:**

Adipocyte-derived hormones play a role in insulin sensitivity and energy homeostasis. However, the relationship between circulating fibroblast growth factor 21 (FGF21), adipocytokines and cold-induced supraclavicular brown adipose tissue (sBAT) activation is underexplored.

**Objective:**

Our study aimed to investigate the relationships between cold-induced sBAT activity and plasma FGF21 and adipocytokines levels in healthy adults.

**Design:**

Nineteen healthy participants underwent energy expenditure (EE) and supraclavicular infrared thermography (IRT) within a whole-body calorimeter at baseline and at 2 hours post-cold exposure. ^18^F-fluorodeoxyglucose (^18^F-FDG) positron-emission tomography/magnetic resonance (PET/MR) imaging scans were performed post-cold exposure. PET sBAT mean standardized uptake value (SUV mean), MR supraclavicular fat fraction (sFF), anterior supraclavicular maximum temperature (Tscv max) and EE change (%) after cold exposure were used to quantify sBAT activity.

**Main Outcome Measures:**

Plasma FGF21, leptin, adiponectin, and tumor necrosis factor alpha (TNFα) at baseline and 2 hours post-cold exposure. Body composition at baseline by dual-energy x-ray absorptiometry (DXA).

**Results:**

Plasma FGF21 and adiponectin levels were significantly reduced after cold exposure in BAT-positive subjects but not in BAT-negative subjects. Leptin concentration was significantly reduced in both BAT-positive and BAT-negative participants after cold exposure. Adiponectin concentration at baseline was positively strongly associated with sBAT PET SUV mean (coefficient, 3269; *P* = 0.01) and IRT Tscv max (coefficient, 6801; *P*  = 0.03), and inversely correlated with MR sFF (coefficient, −404; *P*  = 0.02) after cold exposure in BAT-positive subjects but not in BAT-negative subjects.

**Conclusion:**

Higher adiponectin concentrations at baseline indicate a greater cold-induced sBAT activity, which may be a novel predictor for sBAT activity in healthy BAT-positive adults.

**Highlights:**

A higher adiponectin concentration at baseline was associated with higher cold-induced supraclavicular BAT PET SUV mean and IRT Tscv max, and lower MR supraclavicular FF. Adiponectin levels maybe a novel predictor for cold-induced sBAT activity.

The prevalence of cardiovascular disease, dyslipidemia, and type 2 diabetes is higher among obese compared with lean adults. With over 650 million obese adults worldwide, more needs to be done to curb this pandemic. An emerging strategy involves brown adipose tissue (BAT), which is increasingly being studied since the rediscovery of functional BAT in human adults (1–3). BAT is an intriguing target as it dissipates energy in the form of heat via non-shivering thermogenesis (NST) and contributes to energy expenditure (EE).

Cold exposure is a strong stimulus for BAT activation, and following activation, EE is increased (4). Cold-induced supraclavicular BAT (sBAT) activity can be quantified via positron electron tomography (PET) scans in adults. In our previous findings, we have showed that EE was significantly increased after cold stimulus in BAT-positive subjects (5). The thermogenic features of BAT make it an attractive target for tackling obesity (6). Both experimental and clinical studies have found an inverse relationship between BAT activity and adiposity-related measurements, such as body mass index (BMI) and body fat (7, 8). Activated BAT preferentially oxidizes lipids for fuel and it also utilizes glucose as a metabolic substrate. BAT might thus be explored therapeutically for its anti-obesity, lipid-lowering, and glucose-lowering effects (9, 10).

Cold-induced NST heat production is mediated by the sympathetic nervous system (SNS), which releases norepinephrine to activate BAT (11). In humans, the heat released from activated BAT can be detected via infrared thermography (IRT) (12). SNS activity can be measured by urine or plasma catecholamine concentrations (13). Plasma norepinephrine has been shown to increase during cold exposure in humans (14). However, the relationship between plasma norepinephrine levels and cold-induced BAT activity in healthy adults still remains obscure even though adipose tissue function and SNS activity are tightly interconnected. In healthy individuals, plasma norepinephrine concentrations are not different between those with detectable versus undetectable cold- induced brown fat activation (15). Brittany et al reported that urinary norepinephrine concentrations were negatively associated with cold-induced BAT activity (16). Therefore, whether SNS activity is associated with cold-induced BAT activity still needs further exploration.

White adipose tissue (WAT) has an important role in maintaining energy homeostasis which are mediated by adipose-derived hormones referred to as adipokines (17). Fibroblast growth factor-21 (FGF21) was originally discovered in 2000 as a new member of the FGF superfamily expressed by the liver (as a hepatokine) (18) and the muscles (as a myokine), and it has important effects on energy balance, lipid metabolism, and glucose metabolism (19). FGF21 is also an adipokine since it is also synthesized and released from WAT (20). It shows complementary actions to adiponectin (21). Cold exposure has been reported to increase circulating levels of FGF21 (22), and FGF21 levels are positively correlated with BAT activity during acute cold exposure (23). Leptin, another adipocyte-derived hormone, elicits potent effects on energy homeostasis by suppressing food intake and stimulating EE, particularly through its effects on BAT thermogenesis via the hypothalamic level (24, 25). Adiponectin is an adipocyte-derived hormone that plays a key role in insulin sensitivity and energy homeostasis. Adiponectin protects against obesity-related metabolic complications. Chronic cold exposure–induced adiponectin accumulation in subcutaneous fat is essential for its browning properties in mice models (26). Tumor necrosis factor (TNF) is an adipokine that is elevated in states of obesity and inflammation (27). TNF not only induces insulin resistance by direct interference with the insulin signaling but also by lowering adiponectin (28).

To further understand the role of adipokines and cold-induced sBAT activity, we evaluated the relationship between cold-induced sBAT activity by quantifying sBAT PET standardized uptake value (SUV) mean, magnetic resonance (MR) supraclavicular fat fraction (sFF), IRT supraclavicular maximum temperature (Tscv max), EE change, and circulating adipokines (FGF21, leptin, adiponectin and TNFα) in healthy individuals. We found that plasma adiponectin concentration at baseline exhibits a strong relationship with cold-induced sBAT activity and thus it might serve as a predictor for sBAT activity in BAT-positive individuals.

## Materials and Methods

### Subjects

This is a secondary analysis of data acquired from participants in a protocol that investigated BAT activation and EE by cold and capsinoids stimulation (NCT02964442). A total of 20 individuals were recruited in this study. One participant had difficult venous access and was excluded. Therefore, a total of 19 participants with all the blood parameters were included in the current analysis. Nineteen participants had measurements of sBAT PET SUV mean and MR sFF via PET/MR imaging after cold exposure for approximately 2 hours, and IRT Tscv max, EE change, and plasma adipokines before and after 2 hours of cold exposure. Data from participants with sBAT PET SUV mean ≥ 2 were classified as BAT-positive subjects, as described in detail in our previous paper (5). The study was conducted according to the ethical guidelines of the Declaration of Helsinki, and all procedures were approved by the Domain-Specific Review Board of National Healthcare Group, Singapore (2015/00715). Written informed consent was obtained from all subjects before participation.

### Clinical measurements

Body fat was measured by dual energy x-ray absorptiometry (DXA) (QDR 4500A, fan-beam densitometer, software version 8.21; Hologic, Waltham, MA). Screening fasting blood samples were sent to the National University Hospital, Singapore referral laboratory for biochemical assessment of liver, renal and thyroid function. Fasting and 2-hour postintervention blood samples during the whole-body calorimetry (WBC) session were collected from venous sampling. Plasma concentration of insulin was measured using immunoassay analyzer (Cobas e411, Roche). Plasma concentrations of high-density lipoprotein (HDL) cholesterol, triglycerides, and glucose were measured using a clinical chemistry analyzer (Cobas e311, Roche). Plasma FGF21 was measured using the Quantikine ELISA Kit (R & D System, Minneapolis, MN). Plasma adiponectin concentration was measured using a human adiponectin ELISA Kit (Abcam, Cambridge, UK). Plasma leptin and TNFα were measured using the MILLIPLEX MAP Human Metabolic Hormone Magnetic Bead Panel (Merck, MO).

### Energy expenditure and infrared thermography temperature measurements

Subjects were seated in an upright posture on a chair, with their heads positioned neutrally and arms adducted. A thermal imaging camera (FLIR T440, FLIR Systems, Sweden) was placed in front of the subject and positioned 1 meter from the subject’s face. After a 10-minute acclimatization period, baseline thermal images and baseline resting metabolic rate (RMR) were measured over 45 minutes. Subjects were then asked to wear the cooling vest inside the WBC chamber for a 2-hour measurement. The cooling vest was wrapped around the torso, leaving the anterior supraclavicular (SCV) region exposed. All IRT video recordings were acquired over a standardized recording period of 1 second (30 frames per second), whereby anterolateral views of the SCV bilaterally were captured. The thermal images were recorded every 15 minutes for 2 hours. The subjects were requested to remain as still as possible, with their shoulders apposed squarely against the back of the chair to minimize movement within the image frames during thermal video recordings. Thermal imaging was performed in the same room as the WBC EE measurements, at a constant ambient temperature of 24°C. RMR and change in EE during the 2-hour intervention were calculated based on methodology established in a previous study (29). Anterior SCV temperature is defined as Tscv, and the maximum temperature of Tscv (Tscv max) was used for the analysis.

### Supraclavicular BAT PET SUV mean, MR sFF, and abdominal fat volume measurements

The participants arrived at the Clinical Imaging Research Centre in a fasting state. After 1 to 2 hours of mild cold exposure (~14.5°C) via a cooling vest (Cool58 Polar Product), the participants received an intravenous injection of ^18^F-fluorodeoxyglucose (^18^F-FDG) (3 mCi) and continued wearing the cooling vest for another 20 minutes until entering the scanner. All MR and PET scans were performed by using a hybrid MR-PET system (Biograph mMR, Siemens Healthcare, Erlangen, Germany) for 80 minutes. A 3D prototype multipoint (10 echoes) Dixon sequence was used for the water-fat imaging at the start and end of the scan with following parameters: repetition time (TR), 15 ms; 10 echo times (TEs), 1.23, 2.46, 3.69, 4.92, 6.15, 7.38, 8.61, 9.84, 11.07 and 12.30 ms; flip angle (FA), 4°; field of view (FOV), 384 × 384 mm; matrix size 192 × 192; readout mode, bipolar; 112 slices with 2 mm thickness. A multistep adaptive nonlinear fitting approach was utilized to quantitate the MR sFF map (30). The sBAT depots were manually segmented based on anatomical information in multiple slice images of registered MR and PET images using ITK-SNAP under the close guidance of an experienced clinical radiologist (31). A lower threshold of 40% of sFF values was used to exclude the muscle and bone marrow prior to computation of mean MR FF and PET SUV mean in the segmented sBAT region (32, 33). The cutoff value of sBAT PET SUV mean for categorizing subjects into BAT-positive and BAT-negative groups was 2.0, which has also been applied in other clinical studies (4, 34–36).

MR images of abdominal fat were acquired using a 2-point Dixon sequence (TR = 5.49 ms, TE1 = 2.46 ms, TE2 = 3.68 ms, slice thickness = 3 mm, matrix size = 320 × 240) during a breath hold of 18 to 20 seconds. Abdominal fat was quantified from 80 axial slices from the fat-only image between the L1 and L5 lumbar vertebrae. A fully automated graph theoretic segmentation algorithm was used to separate and quantify the subcutaneous (SAT) and visceral adipose tissue (VAT) depots, and level sets based algorithm separated the deep (DSAT) and superficial (SSAT) subcutaneous adipose tissue depots (37).

### Statistical analysis

Differences between BAT-positive and BAT-negative were assessed using the Student *t* test. Paired *t* tests were used to compare the differences in the concentrations of blood adipokines between baseline and 2 hours after cold stimulation. Spearman correlations were used to assess relationship between variables after adjusting for sex. The linear mixed effects model was used to evaluate the main effects of treatment (cold exposure), sex of BAT-positive participants (female and male), and their interactions while controlling for the baseline measurements. Regression results for “baseline” and “change from baseline” analysis were adjusted for sex, body fat percentage, and HOMA-IR. Regression results for the 120 min cold exposure analysis was adjusted for baseline value, gender, body fat percentage, and homeostatic model assessment for insulin resistance (HOMA-IR). Data are presented as means ±  standard error of the mean (SEM), unless otherwise stated. A *P* value ≤ 0.05 was considered statistically significant. Statistical analysis was performed by using SPSS software version 23 (IBM SPSS Inc.).

## Results

### Characteristics of the participants

Baseline and metabolic characteristics of the total, BAT-positive participants, and BAT-negative participants are shown in Table 1. As previously reported (5), 12 of 19 healthy participants had positive cold-induced sBAT activation via ^18^F-FDG PET/MR scans with a sBAT PET SUV mean ≥ 2. The data from 19 healthy participants, with 12 BAT-positive and 7 BAT-negative individuals (age 26.9 ± 1.2 and 24.4 ± 1.4 years, respectively), were analyzed. The cohort’s BMI and body fat (%) were 21.7 ± 0.6 kg/m^2^ and 29.7 ± 1.8%, respectively. There was a significant difference in fasting triglycerides level by BAT status (BAT-positive 1.0 ± 0.1 mmol/L vs BAT-negative 0.7 ± 0.1 mmol/L; *P* = 0.04). No significant difference was found in other variables between BAT-positive and BAT-negative subjects.

**Table 1.** Characteristics of Total Participants and Participants With and Without BAT After Cold Exposure

	Total (19)	BAT-positive (12)	BAT-negative (7)	*P* value
Age (years)	26.0 ± 1.0	26.9 ± 1.2	24.4 ± 1.4	0.22
BMI (kg/m^2^)	21.7 ± 0.6	21.7 ± 0.8	21.8 ± 0.9	0.95
Body weight (kg)	62.1 ± 3.0	63.8 ± 4.1	59.2 ± 4.0	0.46
Body fat (%)	29.7 ± 1.8	29.4 ± 2.8	30.1 ± 1.5	0.85
Fat mass (kg)	18.6 ± 1.8	19.2 ± 2.7	17.6 ± 1.0	0.67
SAT (cm^3^)	1868 ± 223	1740 ± 364	2234 ± 221	0.53
VAT (cm^3^)	491 ± 88	499 ± 98	480 ± 168	0.92
SSAT (cm^3^)	1059 ± 96	1000 ± 142	1136 ± 127	0.50
DSAT (cm^3^)	809 ± 133	740 ± 225	898 ± 107	0.57
RMR (kcal/day)	1569 ± 75	1574 ± 88	1561 ± 145	0.94
Glucose (mmol/L)	5.5 ± 0.1	5.5 ± 0.2	5.4 ± 0.07	0.78
Insulin (µIU/mL)	10.0 ± 1.0	10.0 ± 1.4	10.0 ± 1.1	1.00
HOMA-IR score	2.45 ± 0.25	2.48 ± 0.38	2.40 ± 0.27	0.89
Triglyceride (mmol/L)	0.9 ± 0.06	1.0 ± 0.1	0.7 ± 0.1	0.04
NEFA (mmol/L)	0.7 ± 0.03	0.7 ± 0.04	0.7 ± 0.04	0.26
FGF21(pg/mL)	85.7 ± 20.2	88.4 ± 28.5	81.1 ± 27.9	0.87
Leptin (pg/mL)	5041 ± 1103	5555 ± 1686	4159 ± 878	0.56
Adiponectin (ng/mL)	8703 ± 688	8321 ± 673	9359 ± 1524	0.48
TNFα (pg/mL)	3.0 ± 0.2	3.0 ± 0.2	2.9 ± 0.3	0.89

Data presented as mean ± SEM. *P* values represents Student *t*-test between BAT-positive and BAT-negative participants.

Abbreviations: BAT, brown adipose tissue; BMI, body mass index (calculated as body weight [kg] divided by the square of height [m]); DSAT, deep subcutaneous adipose tissue; FGF21, fibroblast growth factor-21, HDL, high-density lipoprotein; HOMA-IR, homeostasis model assessment of insulin resistance (calculated as fasting glucose × fasting insulin divided by 22.5); NEFA, non-esterified fatty acids; RMR, resting metabolic rate; SAT, subcutaneous adipose tissue; SSAT, superficial subcutaneous adipose tissue; TNFα, tumor necrosis factor-alpha; VAT, visceral adipose tissue.

### Relationship of baseline adipokines and body composition

There was a positive trend in the association between fasting FGF21 and BMI (*r* = 0.49; *P* = 0.09) after adjustment for sex, but not with other baseline measurements (Table 2). Fasting leptin was positively associated with BMI (*r* = 0.69; *P* = 0.01), fat mass (*r* = 0.87; *P* < 0.001), fat percentage (*r* = 0.85; *P* < 0.001), SAT (*r* = 0.80; *P* < 0.001), SSAT (*r* = 0.75; *P* = 0.003), DSAT (*r* = 0.81; *P* = 0.001), and REE (*r* = 0.65; *P* = 0.02), while it was negatively associated with HDL (*r* = −0.62; *P* = 0.02) but not with other baseline metabolic measurements including glucose (*P* = 0.90), insulin (*P* = 0.34), and triglyceride (*P* = 0.66) after adjustment for sex. Fasting adiponectin was positively associated with HDL (*r* = 0.58; *P* = 0.04) but not associated with other baseline characteristics we have listed in Table 2. Fasting TNFα concentration was significantly negatively correlated with body fat percentage (*r* = −0.56; *P* = 0.03) and DSAT (*r* = −0.52; *P* = 0.05) after adjusting for sex (Table 2).

**Table 2.** Correlations Between FGF21, Leptin, Adiponectin, TNFα, and Body Composition Blood Markers at Baseline in Total Participants

	FGF21		Leptin		Adiponectin		TNFα	
Measure	Coefficient (*r*)	*P* -value	Coefficient (*r*)	*P* -value	Coefficient (*r*)	*P* -value	Coefficient (*r*)	*P* - value
BMI (kg/m^2^)	0.49	0.09	**0.69**	**0.01**	0.02	0.95	-0.27	0.33
fat mass (kg)	0.41	0.17	**0.87**	**<0.001**	-0.13	0.67	-0.42	0.12
% fat mass	0.38	0.21	**0.85**	**<0.001**	-0.37	0.22	**-0.56**	**0.03**
SAT (cm^3^)	0.43	0.14	**0.80**	**0.001**	-0.18	0.55	-0.46	0.09
VAT (cm^3^)	0.25	0.40	0.54	0.06	-0.18	0.56	-0.08	0.79
SSAT (cm^3^)	0.43	0.14	**0.75**	**0.003**	-0.16	0.60	-0.35	0.20
DSAT (cm^3^)	0.42	0.16	**0.81**	**0.001**	-0.19	0.53	**-0.52**	**0.05**
REE (kcal/day)	0.15	0.63	**0.65**	**0.02**	-0.15	0.62	-0.25	0.37
Glucose (mmol/L)	-0.15	0.63	0.04	0.90	0.12	0.71	-0.01	0.99
Insulin (µIU/L)	0.05	0.88	0.29	0.34	-0.22	0.48	-0.27	0.34
HDL (mmol/L)	-0.31	0.31	**-0.62**	**0.02**	**0.58**	**0.04**	0.38	0.17
Triglyceride (mmol/L)	-0.25	0.41	0.13	0.66	-0.45	0.12	0.16	0.56
NEFA (mmol/L)	-0.27	0.38	0.05	0.88	0.50	0.08	0.34	0.22

N = 19, Correlations were used to assess relationship between variables after adjusting for sex. Significant correlations between variables are shown in bold with corresponding coefficient (*r*) and *P* values adjusted for sex.

Abbreviations: BMI, body mass index (calculated as body weight [kg] divided by the square of height [m]); DSAT, deep subcutaneous adipose tissue; FGF21, fibroblast growth factor-21, HDL, high-density lipoprotein; NEFA, non-esterified fatty acids; REE, resting energy expenditure; SAT, subcutaneous adipose tissue; SSAT, superficial subcutaneous adipose tissue; TNFα, tumor necrosis factor-alpha; VAT, visceral adipose tissue.

### Effects of cold exposure on plasma adipokines concentration

After cold exposure for 2 hours, plasma FGF21, leptin, and adiponectin concentrations were decreased compared with baseline in all participants (Table 3). We separated the 19 subjects based on BAT status (BAT-positive and BAT-negative subjects) to examine the treatment and BAT-status effects (Table 3). For FGF21, there was significant effect for treatment × BAT-status interaction (*P* = 0.04), which means that cold exposure–induced FGF21 decrease was affected by BAT status. However, there were no significant effects for treatment and BAT status. For leptin and adiponectin, there were significant effects for treatment (*P* < 0.01), but there were no significant effects for BAT status and interaction. There were no significant treatment, BAT-status, and treatment × BAT-status effects for TNFα (Table 3). We separated the 12 BAT-positive subjects based on sex (6 women and 6 men) to examine the treatment and gender effects (Table 4). For FGF21, there was significant effect for treatment (*P* = 0.04), but there was no significant effect for treatment × gender interaction (*P* = 0.20). Plasma baseline leptin concentration was slightly higher in female participants compared with male participants but this did not reach significance level (*P* = 0.17). There were significant effects for treatment (*P* = 0.001) and treatment × gender interaction (*P* = 0.05). Adiponectin concentration was slightly attenuated after cold exposure. There were significant effects for treatment (*P* < 0.001) and treatment × gender interaction (*P* = 0.03). There were no significant treatment and treatment × gender effects for TNFα (Table 4). For all the 3 adipokines we have measured, there were no significant gender effects (Table 4).

**Table 3.** FGF21, Leptin, Adiponectin and TNFa Concentrations at Baseline and Post Intervention at 2 Hours After Cold Exposure in All, BAT-Positive, and BAT-Negative Participants

	Total Participants (n = 19)		BAT-Positive Participants (n = 12)		BAT-Negative Participants (n = 7)		Treatment Effects	BAT-Status Effects	Interaction
	Baseline	120 min	Baseline	120 min	Baseline	120 min		*P* -values	
FGF21 (pg/mL)	85.7 ± 20.2	58.4 ± 11.9 **#**	88.4 ± 28.5	51.6 ± 12.9	81.1 ± 27.9	69.9 ± 24.3	0.45	0.61	0.04
Leptin (pg/mL)	5041 ± 1103	3102 ± 767*	5555 ± 1686	3549 ± 1181	4159 ± 878	2335 ± 496	0.001	0.99	0.30
Adiponectin (ng/mL)	8703 ± 688	7867 ± 657*	8321 ± 673	7314 ± 635	9359 ± 1524	8815 ± 1419	0.002	0.37	0.28
TNFα (pg/mL)	3.0 ± 0.2	2.9 ± 0.2	3.0 ± 0.2	3.0 ± 0.3	2.9 ± 0.3	2.7 ± 0.5	0.12	0.65	0.15

Data presented as mean ± SEM. The treatment effects, BAT-status effects, and interactions between treatment and BAT status were tested using linear mixed effects model.

Abbreviations: BAT, brown adipose tissue; FGF21, fibroblast growth factor-21; TNFα, tumor necrosis factor-α.

# *P* < 0.05 compared with baseline in each group participants; *<0.01 compared with baseline in each group participants.

**Table 4.** Summary of Outcomes of Adipokines After Cold Exposure in 12 BAT-Positive Participants

	Female Participants (n = 6)		Male Participants (n = 6)		Treatment Effects	Gender Effects	Treatment × Gender Interaction
	Baseline	120 min	Baseline	120 min		*P* values	
FGF21(pg/mL)	122.0 ± 55.2	63.2 ± 24.7	54.9 ± 8.7	40.1 ± 8.4	0.04	0.29	0.20
Leptin (pg/mL)	7937 ± 2998	4926 ± 2176	3173 ± 1122	2172 ± 803	0.001	0.20	0.05
Adiponectin (ng/ mL)	8770 ± 1329	7299 ± 1231	7871 ± 378	7329 ± 509	<0.001	0.75	0.03
TNFα (pg/mL)	2.7 ± 0.4	2.6 ± 0.3	3.3 ± 0.2	3.4 ± 0.3	0.92	0.16	0.30

N = 12; Values are mean ± SEM; The treatment effects, gender effects and interactions between treatment and gender were tested using linear mixed effects model while controlling for the baseline measurements.

Abbreviations: BAT, brown adipose tissue; FGF21, fibroblast growth factor-21; TNFα, tumor necrosis factor-α.

### Relationship of adipokine concentrations and cold-induced sBAT activity measurements

Cold-induced sBAT activity was measured via PET, MR, IRT, and EE modalities as previously reported (33). We ran a regression model to examine the relationship between the adipokines level at baseline and 120 minutes after cold exposure and the change from baseline with each cold-induced sBAT activity modality we have used previously. Among the 12 BAT-positive subjects, adiponectin concentration at baseline was positively associated with sBAT PET SUV mean (coefficient = 3269; *P* = 0.01) and IRT Tscv max (coefficient = 6801; *P* = 0.03), and inversely correlated with MR sFF (coefficient = −404; *P* = 0.02) but not with EE percentage change in BAT-positive individuals, while adjusting for body fat percentage and HOMA-IR (Table 5). Other things being equal, an approximately 3269 ng/ml higher adiponectin concentration at baseline reflects a one-degree increase in sBAT PET SUV mean post–cold exposure in BAT-positive subjects (Table 5). After controlling baseline adiponectin concentration, adiponectin concentration at 120 minutes was negatively associated with PET SUV mean (coefficient = −976; *P* = 0.03) but not with other modalities in BAT-positive participants. Adiponectin concentration change from baseline was negatively associated with sBAT PET SUV mean (coefficient = −587; *P* = 0.04) but not with other modalities in BAT-positive participants (Table 5). However, FGF21 and leptin concentration did not show any significant correlation with any cold-induced sBAT activity via the detection modalities we have utilized in BAT-positive subjects while adjusting for body fat percentage and HOMA-IR, with the exception that FGF21 at 120 minutes post–cold exposure was negatively associated with MR FF (coefficient = −2.04; *P* = 0.04). TNFα concentrations at 120 minutes and change from baseline after cold exposure were negatively associated with sBAT PET SUV mean (coefficient = −0.54; *P* = 0.03; coefficient = −0.50; *P* = 0.007) in BAT-positive participants. No associations were found between adipokines and cold-induced sBAT activity indicators by the investigational modalities we applied in BAT-negative participants, as shown in the data repository (38).

**Table 5.** Regression Results of Adipocytokines Markers With Imaging Parameters and EE After Cold Exposure in BAT-Positive Participants

	sBAT PET SUV Mean		MR sFF		IRT Tscv max		EE % change	
Measure	Coefficient (β) ± SEM	*P* value	Coefficient (β) ± SEM	*P*-value	Coefficient (β) ± SEM	*P* value	Coefficient (β) ± SEM	*P* value
**Baseline**								
FGF21 (pg/mL)	−1.4 ± 40.4	0.97	0.76 ± 4.82	0.88	−4.4 ± 90.6	0.96	−2.0 ± 5.0	0.70
Leptin (pg/mL)	720 ± 1632	0.67	−63.2 ± 196.6	0.76	3628 ± 3452	0.33	−27.8 ± 206.0	0.90
Adiponectin (ng/ mL)	**3269 ± 978**	**0.01**	**−404 ± 111**	**0.02**	**6801 ± 2431**	**0.03**	285 ± 164	0.13
TNFα (pg/mL)	0.70 ± 0.41	0.13	−0.06 ± 0.05	0.27	1.50 ± 0.93	0.15	0.07 ± 0.06	0.27
**120-min cold exposure**								
FGF21(pg/mL)	10.9 ± 8.4	0.24	**−2.04 ± 0.78**	**0.04**	7.6 ± 21.2	0.73	0.86 ± 1.2	0.48
Leptin (pg/mL)	271 ± 164	0.15	−32.4 ± 19.4	0.15	−262 ± 459	0.59	−2.1 ± 24.3	0.93
Adiponectin (ng/ mL)	**−976 ± 356**	**0.03**	1.3 ± 67.5	0.99	−197 ± 1081	0.86	75.9 ± 38.5	0.10
TNFα (pg/mL)	**−0.54 ± 0.17**	**0.02**	0.03 ± 0.03	0.37	−0.59 ± 0.56	0.34	0.01 ± 0.03	0.68
Change from baseline								
∆ FGF21(pg/mL)	11.7 ± 24.0	0.64	−2.5 ± 2.8	0.40	10.1 ± 54.6	0.86	1.97 ± 2.95	0.53
∆ Leptin (pg/mL)	56.7 ± 508	0.91	−13.8 ± 60.6	0.83	−1268 ± 1035	0.26	5.8 ± 63.3	0.93
∆ Adiponectin (ng/mL)	**−587 ± 236**	**0.04**	26.5 ± 37.4	0.50	−532 ± 698	0.47	34.7 ± 38.1	0.39
∆ TNFα (pg/mL)	**−0.50 ± 0.14**	**0.007**	0.04 ± 0.03	0.20	−0.70 ± 0.45	0.16	0.01 ± 0.03	0.98

N = 12. SUV calculated from PET integrated in 80 minutes after cold exposure; FF from MR at the start of the scan after cold exposure; Tscv max from peak of IRT analysis within 120 minutes in the supraclavicular region and EE percentage change from baseline in 120 minutes after cold exposure. The baseline and change from baseline analysis were adjusted with gender, body fat percentage, and HOMA-IR. The 120-min cold exposure analysis was adjusted with baseline value, gender, body fat percentage, and HOMA-IR. Significant regression between variables are shown in bold with corresponding Standardized Coefficient (β) ± SEM and *P* values.

Abbreviations: BAT, brown adipose tissue; EE, energy expenditure; FGF21, fibroblast growth factor 21; HOMA-IR, homeostatic model assessment for insulin resistance; IRT, infrared thermography; MR, magnetic resonance; PET, positron-emission tomography; sBAT, supraclavicular brown adipose tissue; SEM, standard error of the mean; sFF, supraclavicular fat fraction; SUV, standardized uptake value; TNF, tumor necrosis factor; Tscv max, supraclavicular maximum temperature.

### Relationship of FGF21 and adiponectin in BAT-positive participants

**Figure 1.**
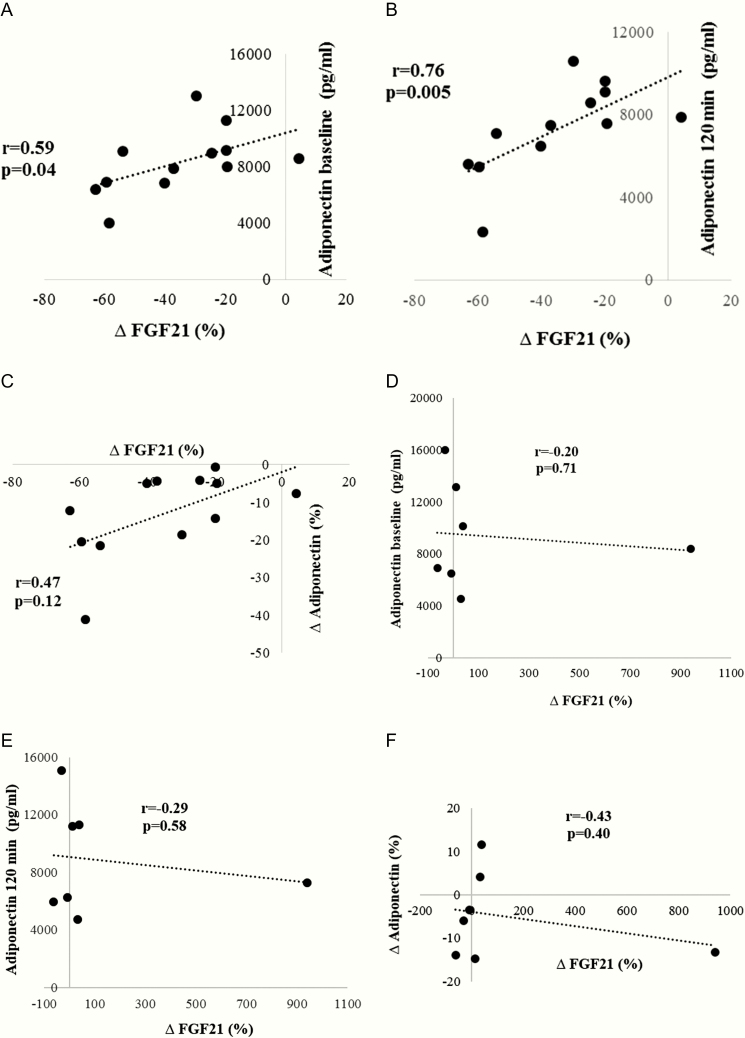
Spearman correlations of plasma FGF21 concentration percentage change (%) and adiponectin concentration at baseline (A, D), 120 minutes after cold exposure (B, E) and percentage change from baseline (C, F) after adjustment for sex in BAT-positive subjects (A, B, C) and BAT-negative subjects (D, E, F). Abbreviation: FGF21, fibroblast growth factor 21.

FGF21 concentration percentage change from baseline was positively associated with adiponectin concentration at baseline (*r* = 0.59; *P* = 0.04), 2 hours after cold exposure (*r* = 0.76; *P* = 0.005), upon adjustment for sex in BAT-positive subjects (Fig. 1A and 1B) but not in BAT-negative participants (Fig. 1D and 1E). There was a trend towards a positive association between FGF21 concentration percentage change from baseline and adiponectin percentage change from baseline (*r* = 0.47; *P* = 0.12) in BAT-positive subjects (Fig. 1C) but not in BAT-negative subjects (Fig. 1F).

## Discussion

The continuing increase in the incidence of obesity and its associated disorders provides much motivation for novel investigation of the fundamental mechanisms of energy regulation. Since 2009, humans have been found to possess functional depots of BAT (1, 3), leading to a revival of interest in BAT as a potential target for increasing EE to combat metabolic disease. Of particular interest are adipokines capable of inducing WAT browning. Adipokines serve an important role in obesity and obesity-related metabolic disorders. There is an extensive cross-talk between white adipocytes and other organs as illustrated by how leptin regulates appetite and energy balance via the hypothalamus, and how adiponectin is involved in regulating EE via activation of BAT thermogenesis (39). In our previous analysis (5), 12 out of 20 subjects defined as BAT-positive had sBAT PET SUV mean ≥ 2 via ^18^F-FDG PET. We have also evaluated cold-induced sBAT activity using MR FF, IRT Tscv max, and WBC EE (33). In this subanalysis, we aimed to investigate the relationship between adipokines and cold-induced sBAT activity measured using different modalities in BAT-positive and BAT-negative subjects. Our key findings were (1) adipokines including FGF21 and adiponectin were attenuated after acute mild cold (∼14.5°C) in those who are BAT-positive; leptin concentration was decreased in both BAT-positive and BAT-negative subjects after acute cold exposure; acute 2-hours cold exposure did not change TNFα level in all the subjects; and (2) adiponectin concentrations at baseline were positively associated with cold-induced sBAT activity markers, including sBAT PET SUV mean and IRT Tscv max, and inversely associated with MR sFF only in BAT-positive subjects.

FGF21 is a cold-induced endocrine activator of brown fat function in humans and has been recognized as a brown fat adipokine, otherwise termed a *batokine* (40). Although the understanding of FGF21 as a major metabolic regulator is rapidly evolving, the scientific appreciation of FGF21 biology is still fragmented and controversial (41). There are reports that FGF21 is expressed in BAT, but its role in metabolism has not been investigated (11, 14, 22). Interestingly, FGF21 produced by the liver as a hepatokine promotes thermogenic activation of brown fat and is dispensable during starvation-induced torpor (23, 24).

Circulating FGF21 level was previously shown to be increased after an extended period of cold exposure, from 24°C to 19°C for 7 hours, and correlated with greater rise in EE, thereby implying that FGF21 may mediate cold-induced metabolic changes (22). However, unlike this long cold exposure described by Lee et al, a shorter cold exposure for 2 hours was not accompanied by a rise in FGF21; instead we observed a decline in plasma FGF21 concentration by 35% ± 6% (*P* = 0.048) in 12 BA-positive participants, consistent with a previous report by the same NIH investigators in BAT-positive participants (40). Plasma FGF21 concentration exhibited a diurnal reduction (22). However, the reduction effect was more pronounced in BAT-positive compared with BAT-negative subjects. Since the participants were of similar age and underwent the study in a similar manner and differed only by BAT status, these results likely support BAT as a source of FGF21, prone to fluctuations during acute cold exposure in humans. Baseline FGF21 expression among BAT-positive people may be higher and this may accentuate the decline in FGF21 upon cold stimulation compared with BAT-negative individuals, whose baseline FGF21 are lower and hence not significantly altered after cold stimulation. Whether FGF21 concentration would be increased during longer cold exposure in humans needs further exploration. Notably, we found a significant inverse association between FGF21 concentration after 2 hours post–cold exposure and MR sFF, which indicated that the greater the degree of FGF21 reduction (the lower FGF21 level at 2 hours of cold exposure), the higher was the BAT activity as shown by a lower MR sFF. These results indicated that cold-induced FGF21 changes may reflect BAT-WAT lipid metabolism.

Leptin levels rise with increased adiposity in human. Human plasma leptin level is highly correlated with BMI and body fat both in men and women (42), consistent with our observations in this study as well. Plasma leptin concentration is negatively correlated with HDL among children (43), a finding similarly shown in the healthy adults of our study. Leptin plays an important role in lipid metabolism. We observed a decrease of plasma leptin in response to activation of the SNS via cold exposure in both BAT-positive and BAT-negative subjects in line with a previous report in healthy women (44). SNS activation suppresses leptin synthesis, thereby decreasing serum leptin in vivo. Leptin plays an important physiological role not only in energy homeostasis as a defense of body fat store but also in the thermoregulation process (45). Leptin levels fall after cold exposure, a response representative of a signal for initiating a broad program of adaptation to starvation (46). This is relevant for our subjects who underwent cold exposure after more than 10 hours in the fasting state. Plasma leptin levels at fasting and 2 hours post–cold exposure did not bear any association with BAT activity markers in BAT-positive subjects after controlling for body fat percentage and HOMA-IR. However, without controlling for body fat, leptin concentration had a strong correlation with EE percentage change from baseline (data not shown). This indicates that plasma leptin levels could be part of the underlying mechanism explaining a more efficient resting EE and change in EE after cold exposure due to the body fat percentage differences. Our results suggested that leptin’s role in thermoregulation involves mechanisms independent of the action of sBAT. Since the physiological role of leptin in BAT has been elusive in humans, further research will be needed to elucidate the mechanism of leptin alteration after BAT activation to better modulate energy balance and adiposity in humans.

Adiponectin, as one of the most abundant adipokines in the body, has been extensively studied in many tissue and organs throughout the body. Adiponectin levels was inversely associated with obesity and insulin resistance in humans (17). We did not see any correlation between body composition and adiponectin levels in healthy lean subjects, unlike the obese subjects (47), probably due to the small sample size and the phenotype of the subjects. The relationship between adiponectin and adiposity was more robust in obese people than lean people. However, we found a significant positive association between fasting adiponectin concentration and fasting HDL level. While speculative, this raises a possible hypothesis that adiponectin might be linked to HDL metabolism. Adiponectin appears to induce an increase in serum HDL, and conversely, HDL can upregulate adiponectin levels (48). The adiponectin-HDL relationship can explain in part the presumed protective role of adiponectin in cardiovascular disease and the adiponectin changes observed after dieting, exercise, and lipid-lowering treatment. The role of adiponectin in SNS activation is somewhat controversial in small rodent studies. Moreover, its role in cold exposure-induced BAT activation has scarcely been explored in humans. Our study is among the first to demonstrate the direct relationship of cold-induced BAT activation on adiponectin regulation in BAT-positive and BAT-negative subjects. In the present study, cold-induced sBAT activation resulted in a lower decline in plasma adiponectin concentration, a finding consistent with previous reports in mice (49) and humans (50).

Adiponectin concentration at baseline was positively correlated with BAT activity measured via PET SUV, MR FF, and IRT Tscv max, which indicated that higher adiponectin concentrations occur in those with higher BAT activity among BAT-positive subjects. Cold-induced adiponectin decline was mitigated by BAT activation. BAT activity is typically measured by imaging ^18^F-FDG uptake in response to cold exposure based on glucose uptake by active sBAT. The positive correlation between adiponectin concentration and ^18^F-FDG uptake may be explained by the stimulation of glucose utilization due to adiponectin (51). Activated BAT also utilizes endogenous fat as fuel and takes up fatty acids from the circulation. Thus, the fat fraction was lower after cold-induced BAT activation. The lower fat fraction indicates a higher BAT activity as reported previously (52). Adiponectin has been shown to increase fatty acid oxidation and reduce circulating free fatty acid and triglyceride levels (53). Consistently, we found that adiponectin concentration was negatively correlated with MR sFF. Recently Qiong et al reported that (54) adiponectin-associated thermogenesis is not essential under normal temperature but is required for fasting and cold-challenged conditions, suggesting that adiponectin is required for maintaining body temperature in a cold environment in a mice model. In line with our study, subjects with higher adiponectin levels had relatively higher Tscv max after cold-induced BAT activation. This implied that the physiological relevance of adiponectin lies in supporting the classical function of BAT by defending the body temperature against hypothermia upon cold exposure. There is a trend of a positive relationship between adiponectin concentration and the EE increase after cold-induced BAT activation but this failed to reach statistical significance, probably due to the small sample size and the relatively larger variation of EE increase of our participants. Chronic cold exposure selectively induces adiponectin accumulation, which is indispensable for WAT browning in mice models (26). However, SNS involvement in adiponectin regulation in blood is somewhat still controversial. In in vivo animal studies, Imai et al (49) reported that cold exposure suppresses serum adiponectin levels after cold exposure for 12 hours in mice. Puerta et al (55) reported that cold (18 hours at 6°C)-induced SNS activation did not significantly affect the serum adiponectin concentrations in rats. Imbeault P et al (56) reported adiponectin plasma concentrations were acutely and significantly increased after 2 hours of cold exposure with food consumption which differs from our study design. Overall, the lower rate of decline of adiponectin concentration with acute cold exposure in BAT-positive subjects probably implied that adiponectin is required for maintaining body temperature under acute cold exposure dependant on BAT. Taken together, serum adiponectin level showed a strong relationship with cold-induced BAT activity only in BAT-positive subjects. However, the feasibility of adiponectin-mediated browning with respect to EE and metabolic homeostasis in humans still needs further investigation.

TNFα has been demonstrated to be elevated in human obesity. It induces insulin resistance, which thus explained the negative correlation between TNFα and body fat and SSAT found in our study (57). Enzo et al (58) reported that TNFα mediated apoptosis of brown adipocytes and triggered defective brown adipocyte function in obesity, indicating that TNFα plays an important role in some aspects of BAT biology. However, plasma TNFα concentration did not change after acute cold exposure in our study. Clearly, more studies on the relationship between TNFα and BAT activity are needed to clarify their mechanistic interactions better.

In our study, we also found that delta FGF21 after 2 hours post–cold exposure significantly correlated with adiponectin concentration at baseline and 2 hours post–cold exposure in BAT-positive subjects. Adiponectin controls systemic glucose and lipid homeostasis in the liver and skeletal muscle in an endocrine manner (59). FGF21 has many functional similarities to adiponectin. These results indicated that BAT may be involved in the FGF21–adiponectin axis and how this in turn controls EE is a matter for further research.

In conclusion, we provide evidence for cold-induced changes in adipokine secretory profile in humans. Adipokines including FGF21 and adiponectin levels were less reduced after acute cold exposure independent of sex in BAT-positive subjects. Leptin levels were reduced after acute cold exposure independent of BAT type and sex. Circulating adiponectin concentration was strongly associated with BAT activity. Adiponectin concentration may henceforth serve as a novel predictor of BAT activity.

## Abbreviations

^18^F-FDG: ^18^F-fluorodeoxyglucose
BAT: brown adipose tissue
BMI: body mass index
DSAT: deep subcutaneous adipose tissue
DXA: dual-energy X-ray absorptiometry
EE: energy expenditure
FGF21: fibroblast growth factor 21
HDL: high-density lipoprotein
HOMA-IR: homeostatic model assessment for insulin resistance
IRT: infrared thermography
MR: magnetic resonance
NST: non-shivering thermogenesis
PET: positron-emission tomography
REE: resting energy expenditure
RMR: resting metabolic rate
SAT: subcutaneous adipose tissue
sBAT: supraclavicular brown adipose tissue
SCV: supraclavicular
SEM: standard error of the mean
sFF: supraclavicular fat fraction
SNS: sympathetic nervous system
SSAT: superficial subcutaneous adipose tissue
SUV: standardized uptake value
TNF: tumor necrosis factor
Tscv max: supraclavicular maximum temperature
VAT: visceral adipose tissue
WAT: white adipose tissue
WBC: whole body calorimetry

## Additional Information

***Disclosure Summary:*** The authors have nothing to disclose.

***Data Availability:*** The datasets generated during and/or analyzed during the current study are not publicly available but are available from the corresponding author on reasonable request.
